# Extra Virgin Olive Oil Reduces the Risk of Non-Alcoholic Fatty Liver Disease in Females but Not in Males: Results from the NUTRIHEP Cohort

**DOI:** 10.3390/nu16193234

**Published:** 2024-09-24

**Authors:** Rossella Donghia, Rossella Tatoli, Angelo Campanella, Giuseppe Losurdo, Alfredo Di Leo, Giovanni De Pergola, Caterina Bonfiglio, Gianluigi Giannelli

**Affiliations:** 1 National Institute of Gastroenterology—IRCCS “Saverio de Bellis”, 70013 Castellana Grotte, Italy; rossella.tatoli@irccsdebellis.it (R.T.); angelo.campanella@irccsdebellis.it (A.C.); giovanni.depergola@irccsdebellis.it (G.D.P.); catia.bonfiglio@irccsdebellis.it (C.B.); gianluigi.giannelli@irccsdebellis.it (G.G.); 2 Section of Gastroenterology, Department of Precision and Regenerative Medicine and Ionian Area, University of Bari, Piazza Giulio Cesare 11, 70124 Bari, Italy; giuseppelos@alice.it (G.L.); alfredo.dileo@uniba.it (A.D.L.)

**Keywords:** non-alcoholic fatty liver disease, extra virgin olive oil, gender

## Abstract

Background: Non-alcoholic fatty liver disease (NAFLD) is the most prevalent chronic liver disease worldwide. One way to resolve this reversible condition is by making dietary changes. Extra virgin olive oil (EVOO) is often associated with an improvement in this disease. The aim of this study was to explore the protective role of EVOO on NAFLD conditions, stratified by gender. Methods: The study cohort included 1426 participants assessed in the second recall of the NUTRIHEP cohort (2014–2018), subdivided by gender and dividing the EVOO intake into quartiles of daily gram intake. Results: The results indicated a protective role of the last quartile of EVOO only for female subjects, OR = 0.43, *p* = 0.02, 0.21 to 0.85 at 95% C.I., whereas in the male sub-cohort, the effect was not statistically significant. Conclusions: The protective role of EVOO is different between genders. This difference has not been explored in the literature, so we conclude that this is one of the few papers in the literature to evaluate a gender difference in the intestinal absorption of humans based on an epidemiological study.

## 1. Introduction

Non-alcoholic fatty liver disease (NAFLD) is the most prevalent chronic liver disease in the world today [1]. Its prevalence has increased significantly in recent years in parallel with the global obesity epidemic, affecting approximately 30% of the adult world population [2]. 

NAFLD is characterized by a fat accumulation in hepatocytes, resulting from an increase in lipid absorption and de novo lipogenesis, and a reduction in fatty acid oxidation and lipid secretion [3]. Reduced fatty acid oxidation and increased cellular lipid content promote chronic oxidative stress in hepatocytes, resulting in the activation of a pro-inflammatory response and cell death mechanisms that contribute to the evolution of NAFLD and its dreaded complications [4,5,6].

NAFLD is defined as a disease continuum from steatosis to non-alcoholic steatohepatitis (NASH), liver fibrosis, cirrhosis, and hepatocellular carcinoma (HCC) [7]. Current scientific evidence also suggests that NAFLD is an important risk factor not only for liver-related complications but also for multi-system diseases such as type 2 diabetes (T2D) [8], chronic kidney disease (CKD) [9], hypertension, dyslipidemia, atherosclerosis, and cardiovascular diseases (CVDs) [7,8]. This is the reason why the presence of NAFLD is strongly associated with an increased risk of all-cause mortality [7,10].

The growing impact of NAFLD on public health dictates an increasingly in-depth study of the factors responsible for its pathogenesis and progression, in order to identify new, effective therapeutic strategies [11,12].

In addition to genetic and epigenetic factors [13,14], it is now known that diet and lifestyle also contribute significantly to the NAFLD incidence, development, and progression [3,15,16]. The risk of developing NAFLD also varies by gender [17]. A recent study shows that women have a lower susceptibility than men to NAFLD, at least until menopause [18]. This result leads to speculation about a possible role of gender in the development of NAFLD, although there has been little work in the literature analyzing the reasons behind this observation [19].

One of the foods that has been shown to be protective against NAFLD, even in high quantities, is extra virgin olive oil (EVOO), whose role in the diet is now widely acknowledged [20] in the literature, although the effect in both genders is still unexplored.

Although there are very few published papers in the literature and some aspects are still completely unexplored, it is well known that there are gender differences in food intake, which can derive from a series of factors, such as physiological, cultural, and social factors. Men generally have a higher calorie requirement than women due to a greater muscle mass, and higher basal metabolism and activity (due to both sports and their jobs). Additionally, studies have shown that men often consume larger food portions than women. This may be influenced by social norms, hunger levels, and individual appetite. They also have a greater preference for salty, protein-rich foods, while women may lean towards sweeter foods. Furthermore, women are more committed to making better food choices than their male counterparts [21].

The aim of our study is to evaluate the effects of extra virgin olive oil (EVOO) consumption on the risk of NAFLD and to observe whether there are differences between genders in terms of liver outcome.

## 2. Materials and Methods

### 2.1. Study Population

The NUTRIHEP study involved a cohort from the municipality of Putignano (Bari) aged ≥18 years in 2005–2006. These subjects were identified from the medical records of family doctors in the same municipality, based on a distribution of gender and age.

The study was structured in two phases, baseline and follow-up 9 years later (2014–2018). A series of useful parameters for the observational study were recorded. All the other details are described in a previously published paper [22].

In this study, we considered a cohort of 1426 subjects, who completed the food questionnaire (FFQ) and had no missing values for the “gender” outcome variable of this study. In fact, only the follow-up cohort was considered, where the information on oil consumption was collected in an optimal manner.

Daily EVOO consumption was categorized in quartiles. NAFLD was diagnosed in subjects with hepatic steatosis on ultrasound [23].

All participants signed informed consent forms after receiving complete information about their medical data to be studied. This study was approved by the Ethical Committee of the Minister of Health (DDG-CE-502/2005; DDG-CE-792/2014, on 20 May 2005 and 14 February 2014, respectively).

In this analysis, only follow-up subjects were included in this analysis, investigating a total of 1426 subjects. In addition, subjects were excluded if they were on medications, such as cholesterol-lowering statins, which could have had an effect on liver metabolism.

NAFLD was diagnosed in subjects with hepatic steatosis on ultrasound, without AFL (alcoholic fatty liver; the cutoffs for AFL were 30 g/day for men and 20 g/day for women [24]) or drug-induced fatty liver disease (e.g., corticosteroids, valproic acid, amiodarone, hepatitis C or B viral infections, or other disorders) [23].

### 2.2. Dietary Assessments

The validated European Prospective Investigation into Cancer and Nutrition (EPIC) FFQ was administered during the visit, and each food item (260 food items) was converted into an average, as EVOO daily intake in grams.

### 2.3. Statistical Analysis

The subjects’ characteristics are reported as mean and standard deviation (M ± SD) for continuous variables and as frequency and percentages (%) for categorical variables.

To test the normality of the distribution and the related choice of non-parametric tests, the Shapiro test was performed.

The Wilcoxon rank sum test was used to evaluate the differences between the gender groups for continuous variables, while the chi-square test or Fisher’s (when necessary) for categorical variables. We estimated a multiple logistic regression model with NAFLD as the dependent variable (yes vs. no) and EVOO quartiles (<2.30 g/die, 2.30–8.10 g/die, 8.10–19.10 g/die, and >19.10 g/die) as predictors. The models were also adjusted for some covariates (age, cholesterol, BMI, smoking, daily alcohol intake, education, and job), and estimated coefficients were transformed into odds ratio (OR) and relative confidential intervals at 95%. Stratified models were built for gender. Margins were calculated from predictions of a previously fitted model at fixed values of some covariates, and after, a marginsplot was built.

To test the null hypothesis of non-association, the two-tailed probability level was set at 0.05. The analyses were conducted with StataCorp. 2023. Stata Statistical Software: Release 18. College Station, TX, USA: StataCorp LLC.

## 3. Results

The NUTRHIEP participants’ characteristics, presented as epidemiological and clinical variables, are reported in Table 1.

The female gender was predominant in our cohort (56.66% vs. 43.34%), with no statistically significant difference in mean age between the two genders (55.37 ± 15.00 vs. 54.48 ± 13.80, *p* = 0.20). More males were single or married/cohabiting than females (*p* < 0.001), and they had a higher average level of education, i.e., secondary school or higher school. Women, however, had higher prevalences in the extreme classes, i.e., no education or only elementary school, as well as graduates and post-graduates, with obvious significant differences between the genders (*p* = 0.04). This is also reflected in the job, where the most prestigious jobs were more prevalent in men (managers and professionals, or crafts, agricultural, and sales workers), while more women were included in the unemployed category (*p* < 0.001).

**Figure 1:**
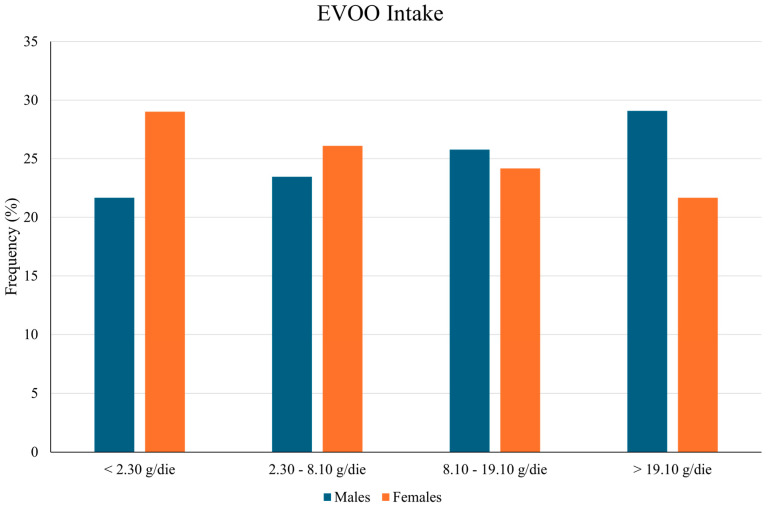
Quartile distribution of EVOO by gender.

Regarding health status and habits, women were less often smokers than men (9.29% vs. 16.21, *p* < 0.001) and had a lower BMI (27.50 ± 5.47 vs. 27.95 ± 4.41, *p* = 0.009). This is obviously related to both systolic and diastolic blood pressure, with statistically significant differences between the genders (118.86 ± 16.31 vs. 124.54 ± 14.61, *p* < 0.0001, 76.32 ± 7.89 vs. 79.86 ± 7.68, *p* < 0.0001). Heart attacks (2.21% vs. 0.66, *p* = 0.01) and liver diseases such as NAFLD (55.66% vs. 44.93%, *p* < 0.001) were statistically significantly more common in men. As for blood parameters, RBC (red blood cells), hemoglobin, MCH (mean corpuscular hemoglobin), MCHC (mean corpuscular hemoglobin concentration), WBC (white blood cells), monocytes (%), eosinophils (%), neutrophils, lymphocytes, monocytes, eosinophils, insulin, HOMA, blood sugar, total bilirubin, GOT (aspartate amino transferase), SGPT (serum glutamic pyruvic transaminase), GGT (gamma-glutamyl transferase), albumin, iron, triglycerides, and ferritin showed significantly higher levels in men (*p* < 0.0001), whereas women had statistically higher levels (*p* < 0.05) of platelets, lymphocytes (%), basophils (%), cholesterol, HDL (high-density lipoprotein), ceruloplasmin, and α1AT (alpha-1-antitrypsin). Worse eating habits were more prominent in men with a higher alcohol consumption (17.41 ± 19.11 vs. 4.22 ± 7.30, *p* < 0.0001), but on the contrary, they consumed more EVOO (17.63 ± 27.39 vs. 13.59 ± 19.29, *p* = 0.002) than women. The lower female intake of EVOO was also demonstrated using the quartile distribution of EVOO (Figure 1):

In addition, gender-stratified association models were developed to evaluate the association between EVOO consumption and the protective role it has against NAFLD (Table 2).

**Figure 2:**
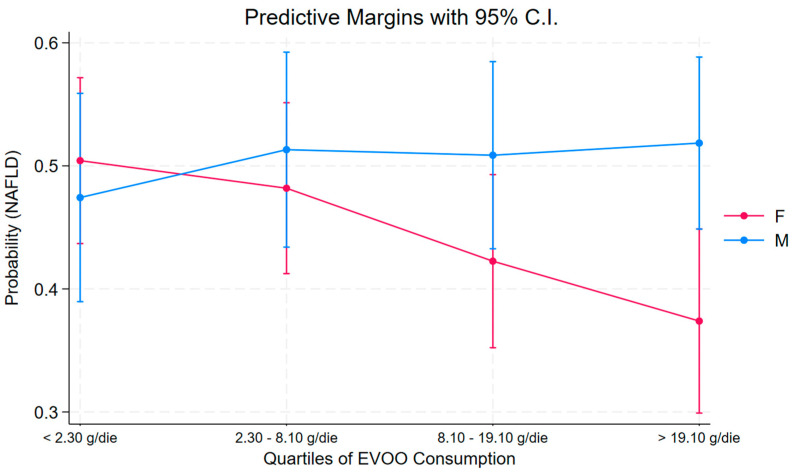
Predictive margins from the model of NAFLD using EVOO quartiles.

In the female sub-cohort, a statistically significant trend was observed, with a protective role of EVOO in the last quartile (OR = 0.43, *p* = 0.02, 0.21 to 0.85 95% C.I.). This trend was not present in the males, where no association was found (Figure 2).

## 4. Discussion

Our study confirms that EVOO is a protective food also against NALFD, but only in females, in a cohort from Southern Italy, where adherence to the Mediterranean diet is still high.

NAFLD is a condition characterized by the accumulation of fat in the liver of people with little alcohol consumption. This condition is becoming increasingly widespread globally, especially in Western countries, and is often associated with obesity, insulin resistance, and the metabolic syndrome [25].

The choice to use the NAFLD definition rather than that of MAFLD to study fatty liver disease not caused by significant alcohol consumption was based on the fact that unlike NAFLD, which relies on a formula that includes age, BMI, diabetes, AST/ALT ratio, platelets, and albumin, there is currently no formula to construct MAFLD. Based on these confusing definitions, NAFLD and MAFLD are considered to overlap [26] and were shown to have high concordance [27]. It should also be noted that NAFLD had much more interesting criteria as they tend to include many more patients. One of the complaints raised with the proposed MAFLD criteria was that they did not consider a substantial percentage of lean patients with respect to NAFLD, and thus normal BMI. In fact, the data indicate that nearly 30–70% of lean NAFLD patients would be missed by MAFLD [28]. We therefore concluded that NAFLD is the best way to describe this type of disease in this cohort, based on the variables measured.

EVOO has attracted attention for its potential benefits in the management of NAFLD due to its unique composition of monounsaturated fatty acids (MUFAs) [29], polyphenols, and other bioactive compounds [30]. It is involved in the improvement of liver enzymes because it can lower the levels of alanine amino transferase (ALT) and aspartate amino transferase (AST) [31], which are often elevated in subjects with liver disease. It can reduce the fat content in the liver because of its action on lipid metabolism, potentially reducing the accumulation of fat in the liver. It also has anti-inflammatory properties due to the presence of polyphenols and other antioxidants. Insulin sensitivity and glucose metabolism can also be improved [32]. Furthermore, its consumption may also be associated with beneficial cardiovascular effects.

The question we should ask ourselves now is how a food like EVOO can have different effects between genders. The answer could lie in physiology, although the literature only contains studies based on murine models. The biological differences between males and females are determined by different gene expressions (a specific set of sex chromosomes and subsequently different fetal development paths) and environmental stimuli, for example, diet. These differences have not been taken into due consideration by the scientific community for socio-cultural reasons, such as education or occupation. In fact, the sphere of nutritional education often considers women separately, exclusively in particular conditions, such as pregnancy or breastfeeding, and when this condition returns to normal. Furthermore, until recently, scientific research was conducted exclusively in male subjects or included both sexes. The end result is that men and women are often considered as “equivalent” [33].

Available data suggest that there is also a clear gender difference in age-related changes in body fat distribution, especially in abdominal adipose tissues, which is accelerated after menopause [34,35,36]. There is a relationship between body fat distribution, plasma levels of sex hormones, and the availability of steroid hormones at the onset of female puberty. These differences are perhaps due to an increased activity of aromatase (a cytochrome P450 enzyme, CYP19, that catalyzes the formation of C18 aromatic estrogens from C19 androgens), especially abdominally [37,38].

About the process of intestinal lipid absorption and transport, much is known but many critical gender-related aspects are unclear. It is well known that female physiology is strongly influenced by the hormonal variations that occur during the menstrual cycle, characterized by notable changes in hormonal concentrations [39].

There are clear differences in gastrointestinal physiology between genders. One example is the different production of gastric acid (GAO) between men and women. Studies have shown that estrogen may play a role in regulating acid secretion and that there are differences in gastric motility between men and women. Also, many studies suggest that women have slower gallbladder emptying than men. These differences may partly explain the greater propensity for females to develop gallstones [40].

The effects of estrogen on gastrointestinal motility are mediated predominantly by nuclear α- and β-receptors and the more recently described transmembrane G protein-coupled estrogen receptors (GPERs), which are found in the myenteric plexus throughout the gastrointestinal tract, including the colon, although such studies are possible exclusively in mouse models [41].

Estrogens may contribute to gender differences by directly influencing lipid metabolism through the suppression of gene expression and the activity of lipoprotein lipase (LPL), the rate-limiting enzyme in the triglyceride metabolism. The modulation of lipolysis through an upregulation of 2-adrenergic receptors could contribute to reducing triglyceride concentrations associated with the modulation of the lipid metabolism [42,43,44,45,46].

## 5. Conclusions

Better knowledge of the possible physiological and molecular mechanisms that are responsible for the different effects of EVOO on a disease condition such as NAFLD could also be important in the study of other foods because it could change the approach toward the treatment of different diseases. Understanding gender differences could lead to the development of dietary plans tailored by gender in order to optimize the beneficial effect of foods.

Furthermore, “Omic” methods will obviously play a fundamental role in this context, with the goal of providing the patient with a more “personalized” treatment.

### Strengths and Limitations

The strengths of the present study include the large cohort and the generalizability of the findings based on a southern Mediterranean population in developing NAFLD. There is currently a lack of data and results in the literature on the effect of this food on different sexes.

Another major selling point is the FFQ, which is the most commonly used dietary assessment method for quantifying food intake. However, it also has some limitations. It would be optimal in a future article to consider not only the individual effects of EVOO between genders in the development of NAFLD but also complex models in order to study the possible interactions between foods and possibly the intake between micro- and macro-nutrients.

## Figures and Tables

**Table 1:** Epidemiological and clinical characteristics of the participants in the NUTRIHEP Study by gender.

Parameters *	Total Cohort (*n* = 1426)	Gender		*p* ^^^
		Female (*n* = 808)	Male (*n* = 618)	
Age (years)	54.87 ± 14.34	54.48 ± 13.80	55.37 ± 15.00	0.20
Civil Status (%)				<0.001 ^ψ^
Single	164 (12.53)	88 (11.91)	76 (13.33)	
Married or Cohabiting	1054 (80.52)	580 (78.48)	474 (83.16)	
Divorced or Separated	30 (2.29)	23 (3.11)	7 (1.23)	
Widower	61 (4.66)	48 (6.50)	13 (2.28)	
Education (%)				0.04 ^ψ^
None	17 (1.30)	11 (1.49)	6 (1.05)	
Elementary School	272 (20.80)	172 (23.27)	100 (17.57)	
Secondary School	389 (29.74)	214 (28.96)	175 (30.76)	
High School	459 (35.09)	238 (32.21)	221 (38.84)	
Degree	58 (4.43)	33 (4.47)	25 (4.39)	
Post-Degree	113 (8.64)	71 (9.61)	42 (7.38)	
Job (%)				<0.001 ^ψ^
Managers and Professionals	110 (7.75)	43 (5.34)	67 (10.89)	
Crafts, Agricultural, and Sales Workers	514 (36.20)	275 (34.16)	239 (38.86)	
Elementary Occupations	193 (13.59)	98 (12.17)	95 (15.45)	
Housewife	153 (10.77)	152 (18.88)	1 (0.16)	
Pensioner	374 (26.34)	183 (22.73)	191 (31.06)	
Unemployed	76 (5.35)	54 (6.71)	22 (3.58)	
Smoker (Yes) (%)	175 (12.29)	75 (9.29)	100 (16.21)	<0.001 ^ψ^
BMI (kg/m^2^)	27.69 ± 5.04	27.50 ± 5.47	27.95 ± 4.41	0.009
Systolic Pressure (mmHg)	121.32 ± 15.85	118.86 ± 16.31	124.54 ± 14.61	<0.0001
Diastolic Pressure (mmHg)	77.85 ± 7.99	76.32 ± 7.89	79.86 ± 7.68	<0.0001
Diabetes (Yes) (%)	92 (6.87)	48 (6.37)	44 (7.50)	0.42 ^ψ^
Heart Attack (Yes) (%)	18 (1.34)	5 (0.66)	13 (2.21)	0.01 ^ψ^
NAFLD (Yes) (%)	707 (49.58)	363 (44.93)	344 (55.66)	<0.001 ^ψ^
Blood Variables				
RBC (M/mcL)	4.94 ± 0.55	4.75 ± 0.46	5.19 ± 0.55	<0.0001
Hemoglobin (g/dL)	13.96 ± 1.49	13.27 ± 1.21	14.86 ± 1.33	<0.0001
Hematocrit (L/L)	41.74 ± 3.77	40.15 ± 3.26	43.80 ± 3.38	<0.0001
MCV (μm^3^)	85.03 ± 8.20	84.98 ± 8.10	85.10 ± 8.34	0.56
MCH (pg)	28.44 ± 2.95	28.11 ± 2.81	28.87 ± 3.07	<0.0001
MCHC (g/dL)	33.40 ± 1.12	33.02 ± 1.02	33.90 ± 1.06	<0.0001
RDW-CV (%)	13.81 ± 1.50	13.83 ± 1.48	13.78 ± 1.53	0.17
Platelets (K/mcL)	233.32 ± 60.96	241.10 ± 58.61	223.15 ± 62.51	<0.0001
WBC (K/mcL)	5.84 ± 1.70	5.62 ± 1.69	6.13 ± 1.66	<0.0001
Neutrophils (%)	57.69 ± 7.80	57.73 ± 7.78	57.64 ± 7.84	0.76
Lymphocytes (%)	31.70 ± 7.25	32.04 ± 7.16	31.26 ± 7.35	0.04
Monocytes (%)	7.31 ± 1.71	7.04 ± 1.58	7.66 ± 1.80	<0.0001
Eosinophils (%)	2.75 ± 1.96	2.64 ± 2.09	2.89 ± 1.77	0.0001
Basophils (%)	0.53 ± 0.31	0.54 ± 0.33	0.51 ± 0.29	0.04
Neutrophils (10^9^/L)	3.41 ± 1.31	3.29 ± 1.31	3.58 ± 1.28	<0.0001
Lymphocytes (10^9^/L)	1.81 ± 0.55	1.76 ± 0.54	1.87 ± 0.55	0.0002
Monocytes (10^9^/L)	0.42 ± 0.14	0.39 ± 0.12	0.47 ± 0.16	<0.0001
Eosinophils (10^9^/L)	0.16 ± 0.13	0.15 ± 0.13	0.18 ± 0.13	<0.0001
Basophils (10^9^/L)	0.03 ± 0.04	0.03 ± 0.05	0.03 ± 0.02	0.48
Insulin (mmol/L)	7.70 ± 5.86	7.22 ± 5.03	8.32 ± 6.75	0.009
HOMA	1.92 ± 2.01	1.75 ± 1.57	2.16 ± 2.46	0.0001
Glycemia (mg/dL)	95.56 ± 17.55	93.30 ± 16.73	98.52 ± 18.16	<0.0001
HbA1c (mmol/mol)	38.10 ± 6.97	37.91 ± 6.55	38.34 ± 7.47	0.88
Total Bilirubin (mg/dL)	0.72 ± 0.38	0.65 ± 0.34	0.81 ± 0.41	<0.0001
GOT (U/L)	22.07 ± 11.62	20.84 ± 13.14	23.68 ± 9.03	<0.0001
SGPT (IU/L)	22.49 ± 16.49	19.91 ± 18.33	25.89 ± 12.95	<0.0001
GGT (U/L)	17.93 ± 14.28	14.86 ± 12.74	21.96 ± 15.18	<0.0001
Alkaline Phosphatase (U/L)	53.13 ± 16.07	53.52 ± 16.80	52.62 ± 15.05	0.51
Albumin (U/L)	4.08 ± 0.27	4.04 ± 0.24	4.15 ± 0.29	<0.0001
Iron (mg/dL)	90.29 ± 31.45	85.04 ± 31.29	97.17 ± 30.33	<0.0001
Cholesterol (mg/dL)	191.57 ± 35.51	194.67 ± 34.26	187.49 ± 36.72	<0.0001
HDL (mg/dL)	50.83 ± 12.71	55.28 ± 12.51	45.00 ± 10.41	<0.0001
Triglycerides (mg/dL)	99.12 ± 69.31	86.56 ± 54.28	115.63 ± 82.30	<0.0001
Ceruloplasmin (mg/dL)	30.94 ± 7.26	33.19 ± 7.66	28.00 ± 5.44	<0.0001
α1AT (mg/dL)	157.73 ± 31.16	161.76 ± 31.69	152.44 ± 29.65	<0.0001
CRP (mg/L)	0.26 ± 0.56	0.27 ± 0.62	0.24 ± 0.46	0.16
Ferritin (ng/mL)	92.16 ± 91.36	56.47 ± 56.74	139.05 ± 105.92	<0.0001
Diet Habits				
Alcohol (g/die)	9.91 ± 15.17	4.22 ± 7.30	17.41 ± 19.11	<0.0001
EVOO (g/die)	15.33 ± 23.21	13.59 ± 19.29	17.63 ± 27.39	0.002
EVOO Quartiles (%)				
<2.30 g	230 (25.30)	145 (28.05)	85 (21.68)	
2.30–8.10 g	227 (24.97)	135 (26.11)	92 (23.47)	
8.10–19.10 g	226 (24.86)	125 (24.18)	101 (25.77)	
>19.10 g	226 (24.86)	112 (21.66)	114 (29.08)	

* Presented as mean and standard deviation (M ± SD) for continuous variables and as frequency and percentage (%) for categorical variables. ^^^ Wilcoxon rank sum test (Mann–Whitney U), ^ψ^ chi-square test or Fisher’s test where necessary. Abbreviations: BMI, body mass index; NAFLD, non-alcoholic fatty liver disease; RBC, red blood cell; MCV, mean corpuscular volume; MCH, mean corpuscular hemoglobin; MCHC, mean corpuscular hemoglobin concentration; RDW-CV, red cell distribution width-coefficient of variation; WBC, white blood cells; HOMA, homeostatic model assessment; HbA1c, hemoglobin A1c; GOT, aspartate amino transferase; SGPT, serum glutamic pyruvic transaminase; GGT, gamma-glutamyl transferase; HDL, high-density lipoprotein; α1AT, alpha-1-antitrypsin; CRP, C-reactive protein; EVOO, extra virgin olive oil.

**Table 2:** Logistic regression ^^^ analysis of NAFLD based on EVOO quartiles, stratified by gender.

Parameter	Female				Male			
	OR	Se (OR)	*p*	95% C.I.	OR	Se (OR)	*p*	95% C.I.
Quartile								
<2.30 g/die [Ref.]	--	--	--	--	--	--	--	--
2.30–8.10 g/die	0.90	0.29	0.73	0.48 to 1.68	1.26	0.49	0.54	0.59 to 2.69
8.10–19.10 g/die	0.59	0.19	0.11	0.31 to 1.12	1.27	0.48	0.53	0.61 to 2.65
>19.10 g/die	0.43	0.15	0.02	0.21 to 0.85	1.34	0.49	0.42	0.65 to 2.73

Abbreviations: NAFLD, non-alcoholic fatty liver disease; OR, odds ratio; se (OR), standard error of OR; 95% C.I., confidence interval at 95%. ^^^ Adjusted for age, cholesterol, BMI, smoking, daily alcohol intake, education, and job.

## Data Availability

The original contributions presented in this study are included in this article. Further inquiries can be directed to the corresponding author.
